# A General Way to Fabricate Chain-like Ferrite with Ultralow Conductive Percolation Threshold and Wideband Absorbing Ability

**DOI:** 10.3390/nano12091603

**Published:** 2022-05-09

**Authors:** Cong Chen, Haitao Dong, Jiayuan Wang, Wen Chen, Denghui Li, Meng Cai, Kun Zhou

**Affiliations:** 1 School of Physics and Electronic Information Engineering, Qinghai Nationalities University, Xining 810007, China; haitao_dong@asia-silicon.com (H.D.); wangjiayuan@163.com (J.W.); chenwen@163.com (W.C.); li15039538994@163.com (D.L.); m13333670730@163.com (M.C.); zkzm0513@sina.com (K.Z.); 2 Asia Silicon (Qinghai) Co., Ltd., Xining 810007, China

**Keywords:** magnetic chain-like material, ferrite, dielectric loss, electromagnetic absorption

## Abstract

The magnetic nanochain-like material has been regards as one of the most promising electromagnetic (EM) absorbing material but remains a challenging. Herein, magnetic chain-like ferrite (included Fe_3_O_4,_ CoFe_2_O_4_ and NiFe_2_O_4_) are successfully produced through a general solvothermal method, using PVP as the structural-liking agent. Experimental results confirm the ultimate sample possess a 3-dimensional chain-like structure which are constructed by numerous ferrite’s nanoparticles with ~60 nm in diameter. Their electromagnetic parameters can be also manipulated by such a chain structure, especially the dielectric loss, where a sharply increases can be observed on within a lower filling ratio. It greatly benefits to the EM absorbing property. In this article, the electromagnetic absorption layer made with a lower content of ferrite possess the excellent electromagnetic absorption ability, where the optimized effective absorption band was nearly 6.4 GHz under a thickness of 1.8 mm. Moreover, the filling ratio is only 30 wt%. Our method for designing of chain-like magnetic material can be helpful for producing wideband electromagnetic absorption in a low filling ratio.

## 1. Introduction

Recently, great achievements are being made in wireless techniques, especially more and more wireless-related electronics have been used in our daily life [1,2,3]. However, the frequently utilization of these electronics will inevitably lead to the serious electromagnetic (EM) radiation or interference, which would not degrade the normal working of neighboring electronics, but also threat human being’s health [4,5]. The exhibition of EM issue has stimulated researchers to produce functional materials, which enabling to absorb the EM energy and then dissipate it into heats [6,7,8]. These functional materials are termed as EM absorbing materials that the mechanism for dissipating the EM energy is via magnetic or dielectric loss ability [9,10]. The key requirement for an exceptionally EM absorber is included wideband, strong absorption, thin thickness etc. [11,12]. Among these candidates, spinel of ferrite (such as Fe_3_O_4_, CoFe_2_O_4_) has attracted a great deal of research interest, owing to the dual magnetic and dielectric loss ability [13,14]. For example, Wang et.al. fabricated a CoFe_2_O_4_ nanoparticle, with a minimum reflection loss value (*RL*) of −9.8 dB under a thickness of 2.8 mm [15]. Zhu and co-workers have developed a hierarchical shaped CoFe_2_O_4_, which possessed a minimum RL value of −17.5 dB within a thickness of 2.5 mm [16]. According to these two cases, it is unfortunate that currently ferrites did not present ideal EM performance, which are specific reflected in the smaller RL value, narrow effective absorption region (frequency region with RL exceeding −10 dB), larger thickness (for commercial application, thickness < 2.0 mm) [17,18]. More importantly, the fill ratio of ferrite is usually higher than 60 wt%, which would result in high cost and excessive weight [19,20]. The in-depth analysis revealed that the EM performance of ferrites are mainly restricted by the low dielectric loss ability [21,22]. To strength the dielectric loss intensity, designing of ferrite-based composites has been an effectively strategy, which were constructed the ferrite with the material than higher in dielectric loss ability [23,24]. These higher dielectric materials are primarily included graphene, multiwall nanotube, metal etc. [25,26,27,28]. As a result, the dielectric loss intensity of ferrite composites would increase significantly, which are helpful for the EM absorption. For example, the *ε*’’ value of original CoFe_2_O_4_ was nearly 1.0, but sharply increased to 4, after decorated with graphene, according to the example of Jason [29]. This method is effectively to dielectric loss, however, the preparation method is complicated, some are involving three or more steps. Meanwhile, due to the exhibition of nonmagnetic component, the integrated magnetic loss ability actually possessed a decreased tendency, thus would weaken the EM absorption ability. Consequently, the electromagnetic performance has improvement a little, but is still insufficient for commercial application.

In this article, herein, we designed a chain-shaped ferrite which using the structural strategy to increase the dielectric loss ability. The as-prepared chain-shaped ferrite (Fe_3_O_4_, NiFe_2_O_4_, CoFe_2_O_4_) were easier to form a 3D network structure after dispersing into the matrix. Once 3D network structure is formed, the dielectric loss ability can be increased sharply, based on the conductive percolation threshold. Such a chain-like sample possess a wideband EM absorption ability under a thin thickness. The method of utilizing structural strategy to formation of chain-shaped ferrite has great significance in making wideband high-performance absorber.

## 2. Experimental Procedure

### 2.1. Materials

Cobalt acetate (Co(Ac)_2_), ferric chloride (FeCl_3_), ferrous chloride (FeCl_2_), Nickel acetate (NiCl_2_) were obtained from Shandong Chemical Reagent Co., Ltd. (Shandong, China). Polyvinylpyrrolidone (PVP, MW58000), ethylene glycol (EG), cyclohexane and glycerol were purchased from Sinopharm Chemical Reagents Co. (Beijing, China). All of the chemical reagents were analytically pure and used without further purification.

### 2.2. Preparation of Chain-Shaped Ferrites

The chain-shaped ferrite was prepared by a solvothermal-process. Typically, 1.5 g PVP, 2.0 mmol FeCl_3_, 1.0 mmol FeCl_2_ are dissolved into solution, containing ethylene glycol (20 mL), and then ultrasonic for 1.0 h. Subsequent, the solution was transferred into a Teflon-lined stainless-steel autoclave and kept at 200 °C for 20 h. After cooled to room temperature, the precipitation can be collected by centrifuge (rotating speed~10,000 rpm), washed with isopropanol, cyclohexane, and ethanol for 6~10 times. Finally, the dried sample was continuous to heat at 300 °C for 2 h, aiming to removal of PVP. The NiFe_2_O_4_ and CoFe_2_O_4_ were prepared via replacing the FeCl_2_ with Co(Ac)_2_) and NiCl_2_.

### 2.3. Characterization and Measurements

The phase compositions of these hybrids are confirmed by powder X-ray diffraction (XRD) patterns, using Cu Kα radiation (λ = 0.154178 nm). Morphologies, especially the chain-like structure are observed by a Field emission scanning microscope (FE-SEM, JEOL JEM-2100, Tokyo, Japan). Fourier transform infrared spectra (FT-IR) was characterized by the Fourier transform infrared spectrometer (VERTEX80, Bruker, Billerica, MA, USA). Magnetization hysteresis loops (M-H) curve was recorded on a vibrating sample magnetometer (VSM, Lakeshore, Model 7400 series, Westerville, OH, USA) at 298 K.

### 2.4. Electromagnetic Parameters

To obtain the electromagnetic parameters, the as-prepared ferrites were homogeneously blended with paraffin wax in the weight ratios of 10~40 wt%. Subsequent, the mixture was pressed into a ring-shaped structure with outer diameter of 7.0 mm and inner diameter of 3.04 mm, respectively. The electromagnetic parameters were analyzed on an E5080A vector network analyzer at 2–18 GHz. Finally, the frequency dependency of reflection loss (*RL*) curve could be gained by inputting the electromagnetic parameters into the below formulas [30,31,32]:

*Z_in_* = *Z_o_*(*μ_r_*/*ε_r_*)^1/2^tanh[*j*(2*πfd*(*μ_r_ε_r_*)^1/2^/*c*)] (1)

*RL*(dB) = 20log|(*Z_in_* − *Z_o_*)/(*Z_in_* + *Z_o_*)| (2)

where *Z_in_* relates to input impedance of the absorber, *f* is the frequency of electromagnetic wave, *d* is the thickness of the absorber, while *c* is the velocity of light. *ε_r_* (*ε_r_*= *ε′*−*jε″*) and *µ_r_* (*µ_r_* = *µ′*−*jµ″*) are the relative complex permittivity and permeability of the absorber.

## 3. Results and Discussion

**Figure 1:**
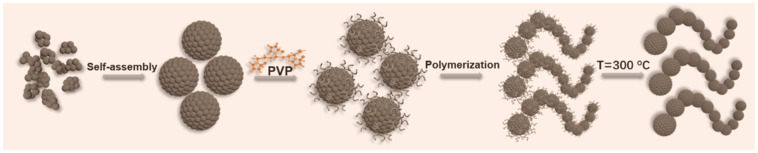
Schematic illustration for the formation procedure of chain-like ferrite.

The chain-like ferries were prepared through a solvothermal route, as see the Figure 1. During the solvothermal procedure, numerous of ferrite nanocrystals would form first and then self-assembly into a nanoparticle. The presence of PVP would adsorb on the surface of nanoparticles and prevent the further growth. Meanwhile, the PVP was constructed by two types of active covalent bonds, knowing as C=O and C–C, respectively. During the solvothermal process, C=O and C=C bonds can be break down and convert into unsaturated –C–O– and –C–C– bonds. These unsaturated activity bonds could continue to link with PVP that adsorbed on neighboring ferrite nanoparticles. Lastly, it would leaded to the chain-shaped structure. Usually, the existed PVP are weaken in electromagnetic absorption, thus needs to be remove. To remove the PVP, the as-obtained samples were processed at 300 °C for 1 h. Relied on such a solvothermal way, three types of chain-like ferrites, included Fe_3_O_4_, CoFe_2_O_4_ and NiFe_2_O_4_ can be made.

**Figure 2:**
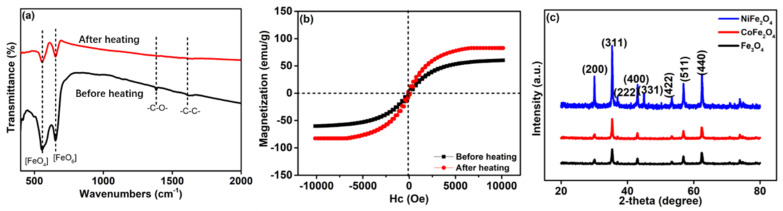
(**a**) FT-IR spectra and (**b**) M–H loops of the chain-like Fe_3_O_4_ sample with and without annealing; (**c**) XRD patterns of chain-like ferrites, included Fe_3_O_4_, NiFe_2_O_4_ and CoFe_2_O_4_.

To make sure the successfully removal of PVP, FT-IR spectra of Fe_3_O_4_ sample was provided in Figure 2a, aiming to observe the changes of covalent bonds. Clearly, without annealing treatment, two Fe-O peaks can be observed at 553 and 655 cm^−1^ which are ascribed to the FeO_4_ (550 cm^−1^) and FeO_6_ (670 cm^−1^) [33,34]. It suggests the spinel phase of Fe_3_O_4_. In addition, another two peaks at 1389 and 1620 cm^−1^ are belonging to C–O and C–C, which are original from the PVP. After annealed at 300 °C, C-based peaks are entirely disappeared, which may be due to the decomposition of PVP.

The magnetization properties were compared by the VSM at room temperature. Figure 2b shows the magnetic hysteresis loops (M–H). The chain-like sample after treated at 300 °C has a higher magnetization value of 84.3 emu/g than the sample without annealing, which attributing to the removal of nonmagnetic PVP. Considering the evidences of FT-IR and M-H loops, the adsorbed PVP can be totally decomposed after conducting annealing treatment. The phase composition of these heated chain-like ferrites are characterized by XRD patterns. As shown in Figure 2c, these diffraction peaks at 2θ = 30.1, 35.3, 37.1, 42.9, 45.3, 53.4, 57.0 and 62.8 ^o^ are corresponding to (200), (311), (222), (400), (331), (422), (511) and (440) crystal planes of spinel Fe_3_O_4_ (JCPDS: card no: 75–1609). In comparison with Fe_3_O_4_, the diffraction peaks of CoFe_2_O_4_ and NiFe_2_O_4_ present a slight right shift, which is due to varied unit cell volume after dotted by Co or Ni.

**Figure 3:**
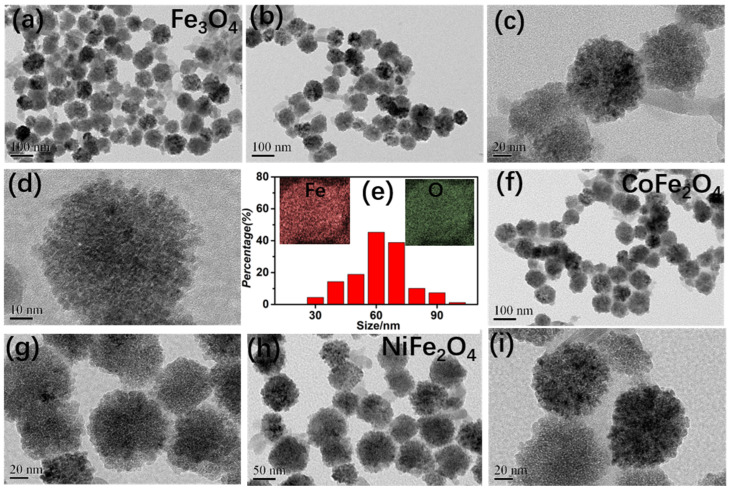
Typical TEM images and size distribution of chain-like ferrites: (**a**–**e**) Fe_3_O_4_; (**f**–**g**) CoFe_2_O_4_; (**h**,**i**) NiFe_2_O_4_.

The chain-like structures were investigated by the TEM images, as showed in Figure 3. In Figure 3a–d, the Fe_3_O_4_ nanoparticles closely contact well with each other and resulting in distinct chain-shaped structure. The average sizes of Fe_3_O_4_ nanoparticles are approximately 60 nm, as statists in Figure 3e. The inserted element mappings reveal that Fe and O elements are evenly distributed in each nanoparticle. Similarly, CoFe_2_O_4_ and NiFe_2_O_4_ both possess the same chain-like structure, as depicted in Figure 3f–i. Meanwhile, the presented nanoparticles have same shapes. Based on TEM images, one conclusion can be made that this method has been proven effectively to form various ferrite nanochains.

**Figure 4:**
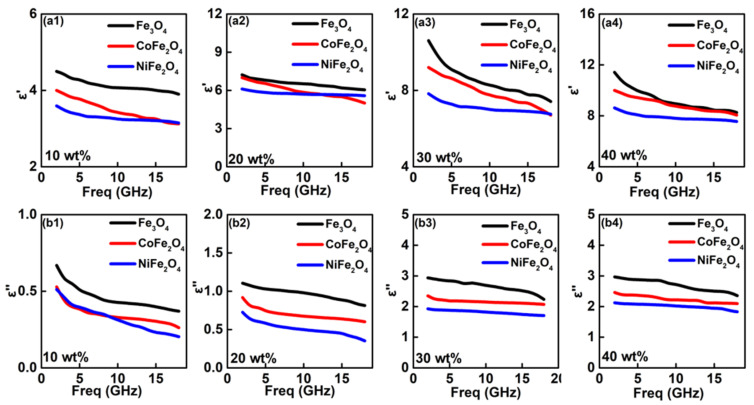
Frequency dependency of permittivity values for the absorption layer with various contains of ferrites: (**a1**) *ε*′-10wt%; (**a2**) *ε*′-20wt%; (**a3**) *ε*′-30wt%; (**a4**) *ε*′-40wt%; (**b1**) *ε*″-10wt%; (**b2**) *ε*″-20wt%; (**b3**) *ε*″-30wt%; (**b4**) *ε*″-40wt%.

Subsequent, the as-prepared ferrites were homogeneous mixed with paraffin in various certain weight ratio, which was used to test the EM parameters. Figure 4 shows the measured permittivity parameters. It is well-known that permittivity values contain two parts, namely real and imaginary part of permittivity value (*ε*′, *ε*″), which are account for the electrical storage and dielectric loss capability, respectively [35,36]. In Figure 4(a1–a4), we observe that all *ε*′ values exhibit the decreased tendency without any remarkable fluctuation. Meanwhile, *ε*′ linearly increases significantly as enhancing the weight ratio of ferrite. It is interesting that exceeding 30 wt%, *ε*′ values become slowly increases. Among these ferrites filling absorption layers, Fe_3_O_4_ has the largest *ε*′ value at whole 2~18.0 GHz. For example, the absorption layer containing 10 wt% of Fe_3_O_4_ has a *ε*′ value about 4.3~3.9, which is greater than CoFe_2_O_4_ (3.8~3.1) and NiFe_2_O_4_ (3.4~3.1), respectively. At 30 wt%, Fe_3_O_4_ still reaches the largest *ε*′ value of 9.4–7.4. The *ε*″ value as a function of frequency are shown in Figure 4(b1–b4). With regarding to the *ε*″ value, similar phenomenon can be observed, and two conclusions are summarized as follows:

1. A higher content of ferrite would lead to the strong dielectric loss ability. Besides, the distinct enhancement of *ε*″ can be observed at ferrite weight regions of 10~30 wt%, but slowly increases between 30~40 wt%.
2. Fe_3_O_4_ is easier to present the strongest dielectric loss behavior than CoFe_2_O_4_ and NiFe_2_O_4_.

To reveal the varied permittivity, the conductive percolation has been used in this article. As we known, *ε*′ and *ε*″ are actually highly associated with their relaxation polarization and conductive loss ability [37,38]. Of particularly note, polarization relaxation at GHz is mainly ascribed to the dipole polarization of ferrite and the interfacial polarization from the interface between ferrite nanoparticles and paraffin wax [39,40]. Either dipole and interface occurs, it would affect *ε*′ and *ε*″ value both, which two typically physical phenomena can be observed, that is, sharply decreased *ε*′ value since the frequency dispersive effect, and dielectric resonance peak in *ε*″ [41]. Concerning the frequency dispersive, the plots of *ε*′ versus *ε*″ will turn to be a single semicircle, normally denoted as the Cole-Cole semicircle, according to the classic Debye-theory. In details, the relative complex permittivity can be drawn as follow [42,43]:

(3) $$\varepsilon_{r}=\varepsilon_{\infty }+\frac{\varepsilon_{s}-\varepsilon_{\infty }}{1+j2\pi f\tau }=\varepsilon^{\prime }-j\varepsilon^{\prime \prime }$$

where *ε*_s_, *ε*_∞_, *τ* are static permittivity, relative dielectric permittivity at high-frequency limit, and polarization relaxation time, respectively. After the separation of real and imaginary parts, gives:

(4) $$\varepsilon^{\prime }=\varepsilon_{\infty }+\frac{\varepsilon_{s}-\varepsilon_{\infty }}{1+{\left(2\pi f\right)}^{2}\tau^{2}}$$

(5) $$\varepsilon^{\prime \prime }=\frac{2\pi f\tau (\varepsilon_{s}-\varepsilon_{\infty })}{1+{\left(2\pi f\right)}^{2}\tau^{2}}$$

Based on the Equations (4) and (5), the *ε*′-*ε*″ can be expressed as above:

(6) $${\left(\varepsilon^{\prime }-\varepsilon_{\infty }\right)}^{2}+{\left(\varepsilon^{\prime \prime }\right)}^{2}={\left(\varepsilon_{s}-\varepsilon_{\infty }\right)}^{2}$$

**Figure 5:**
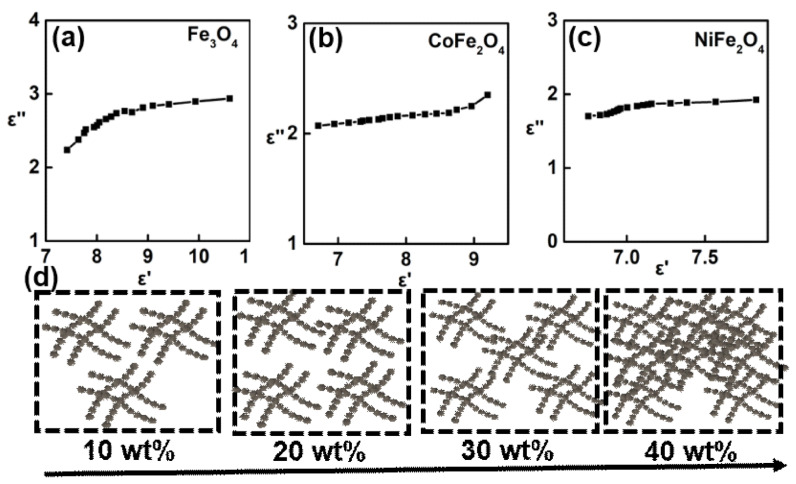
(**a**–**c**) Cole-Cole curves for the 30 wt%-ferrite-paraffin wax composite; (**d**) schematic illustration of the relationship between weight ratios and conductive loss.

According to Equation (6), each Cole-Cole semicircle is corresponding to one Debye relaxation process. Taking the 30 wt% of ferrites as cases, they did not present obviously semicircles, as shown in Figure 5a–c. In this case, it can be deduced that the ferrite-paraffin wax material systems are weakening in polarization. Hence, the dielectric loss is mainly original from the conductive loss. The effect of structure on the conductive are illustrated in Figure 5d. Dispersing a low content of ferrite into the paraffin wax would result in various discontinuous conductive network. Hence, the conductive loss ability is very weakening, owing to the limited transport of electrons. When increases to a certain value, these discontinues are turned to connect with each other and forming a continues conductive network, which greatly enhances the *ε*′ and *ε*″ both. Usually, the weight value for fabricating continuous conductive network was denoted as percolation threshold [44]. Before reaching the percolation threshold, *ε*′ and *ε*″ values increases as rising the weight ratio. Once beyond the percolation value, *ε*′ and *ε*″ possess slowly increasing tendency. In our case, the percolation threshold value of ferrite is estimated to be 30 wt%, which are almost a half of current advances [45,46]. The percolation threshold is not only related to the physical performance of filler, but also influenced by the nanostructure. In this article, the chain-like structure of ferrite can be regarded as the benefited nanostructure, so that a lower filling ratio is enough to form such a continuous conductive network. Hence, these ferrite filling absorption layer enables a good dielectric loss under a relative low filling ratio. Among these ferrites, Fe_3_O_4_ with the highest *ε*″ value which may due to the strongest electron hoping between Fe^3+^ and Fe^2+^ [47].

**Figure 6:**
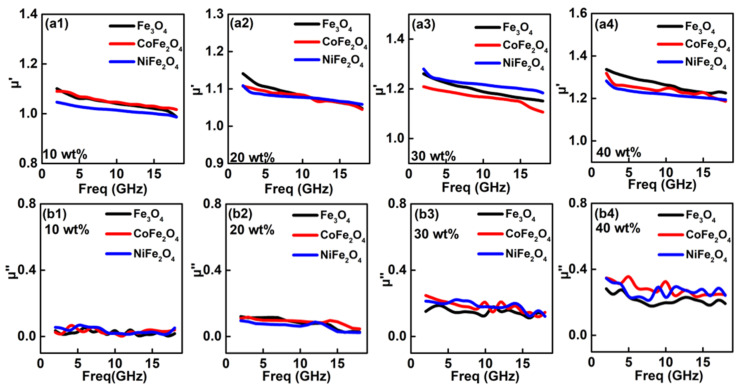
Frequency dependency of permittivity values for the absorption layer with various contains of ferrites: (**a1**) *μ*′-10 wt%; (**a2**) *μ*′-20 wt%; (**a3**) *μ*′-30 wt%; (**a4**) *μ*′-40 wt%; (**b1**) *μ*″-10 wt%; (**b2**) *μ*″-10 wt%; (**b3**) *μ*″-30 wt%; (**b4**) *μ*″-40 wt%.

The permeability values are investigated in Figure 6, which contains real and imaginary part of permeability values (*μ*′ and *μ*″). Generally, the *μ*′ and *μ*″ values are standing for the storage and dissipation capability of magnetic field, respectively [48]. At 10–20 wt%, *μ*′ values of these ferrites filling absorption layer are only a tiny bigger than 1.0. After increasing to 30 wt%, these *μ*′ values are ranging in 1.25~1.15 and simultaneous possessing a decreased tendency. Up to 40 w%, *μ*′ values have a distinct improvement and all of *μ*′ values are larger than 1.25. But overall, *μ*′ of either Fe_3_O_4_, CoFe_2_O_4_ or NiFe_2_O_4_ has a little difference in *μ*′ values, which were attributed to the nearly magnetization behaviors. Because of similar crystal structure and magnetization, their present approximately magnetic loss ability.

**Figure 7:**
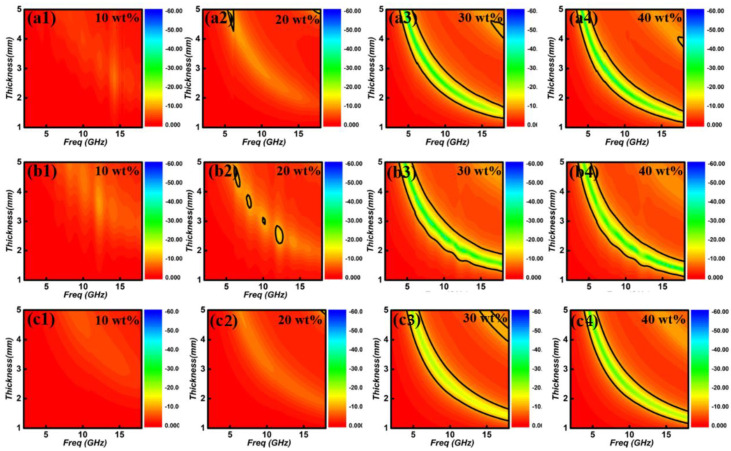
3D RL maps of Fe_3_O_4_ (**a1**–**a4**), CoFe_2_O_4_ (**b1**–**b4**,**c1**–**c4**) NiFe_2_O_4_/paraffin wax composites with different thickness in the frequency range of 2~18.0 GHz.

**Figure 8:**
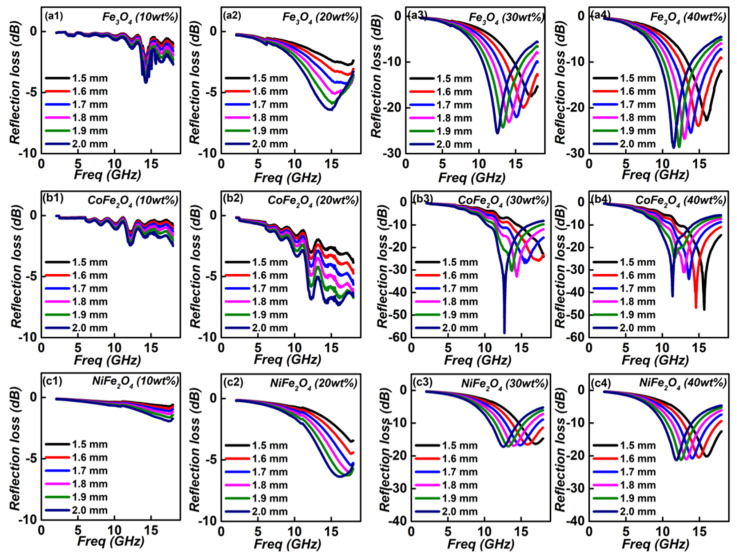
2D RL maps of Fe_3_O_4_ (**a1**–**a4**), CoFe_2_O_4_ (**b1**–**b4**,**c1**–**c4**) NiFe_2_O_4_/paraffin wax composites with different thickness in the frequency range of 2–18.0 GHz. Thickness ranges in 1.5–2.0 mm.

The reflection loss values (RL) values of these samples can be obtained via coaxial-line method. One can see that ferrite filling absorption layer shows the poor electromagnetic absorption, which the reflection loss value is as bigger as −10 dB (regarding as the standard absorption value) at entirely thickness region (Figure 7). But concerning the thickness, the exceptionally EM absorption performance is requested to be thin thickness (<2.0 mm). In order to give a visual effect of the thickness and absorption performance, the RL values of the ferrites filling absorption layer with a thickness region of 1.5~2.0 mm were converted into 2D maps, as presented in Figure 8. When containing 10 wt% of ferrites, their minimum RL values are greater than −5 dB, suggesting the poor EM absorption performance. Increasing to 20 wt%, their minimum RL values decreases, but still larger than −10 dB, thus can’t be used. Significantly enhancement can be found for the absorption layer with a filling ratio of 30 wt%. Specifically, the minimum reflection loss value of −25.5 dB can be gained within a thickness of 2.0 mm. At 1.8 mm, the frequency region with RL < −10 dB can reach maximum (6.4 GHz, 11.6~18.0 GHz), showing desirable wideband absorption ability.

In comparison with Fe_3_O_4_, the minimum RL value of CoFe_2_O_4_ absorption layer equals to −57.8 dB with a matched thickness of 2.0 mm. Meanwhile, the maximum effective absorption region is estimated to be 6.2 GHz under identical 1.8 mm. As for the NiFe_2_O_4_, the optimized RL value and effective absorption region respective −17.1 and 4.2 GH, and corresponding thickness are 2.0 and 1.8 mm. But continues increases to 40 wt%, minimum RL value and effective absorption band do not enhance significantly. To comprehensively consider the bandwidth, thickness and filling ratio, the absorption layer made with 30% of Fe_3_O_4_ would be the optimized electromagnetic performance. Compared with the ferrite-based EM materials in recent advances (listed in Table 1), it is clearly the Fe_3_O_4_-paraffin wax exhibited the excellent within a lower filling ratio.

## 4. Conclusions

To summary up, magnetic chain-like ferrites (such as Fe_3_O_4_, CoFe_2_O_4_ and NiFe_2_O_4_) constructed by 60 nm of nanoparticles have been prepared by a facile solvothermal route. The as-prepared chain-like structure are highly favoring for the permittivity values under a relatively low filling ratio. The results indicate that *ε*″ value of the absorption layer filling with 30% were beyond 2.5. Meanwhile, such a magnetic ferrite possessed moderately magnetic loss ability. Owing to the dielectric and magnetic loss behavior, a wideband absorption region of 6.4 GHz under a thinner thickness of 1.8 mm. The EM absorption mechanism can be obtained at a lower filling ratio has been in-depth investigation, which are greatly benefits to the chain-like structure.

## Figures and Tables

**Table 1:** Ferrites based electromagnetic absorption performance according to recent advances.

Samples	Thickness (mm)	Filler Ratio (wt%)	*Mini. Reflection Loss Value (dB)*	*Effective Absorption Region (GHz)*	Ref.
NiFe_2_O_4_	2.7	20	−70.7	3.5	[49]
Fe_3_O_4_/C	2.1	50	−54.6	6.0	[50]
G–4	1.5	50	−43.9	6.0	[51]
NiCo_2_O_4_/CNTs	4.0	30	−45.1	4.0	[52]
Fe_3_O_4_/MWCNTs	2.0	50	−63.6	3.0	[53]
Co_7_Fe_3_	2.0	20	−78.4	6.7	[54]
Fe_3_O_4_@NPC	3.0	30	−65.5	4.5	[55]
Chain-like-Fe_3_O_4_	1.8	30 wt%	−25.5	6.4 GHz	This work

## Data Availability

Data can be available upon request from the authors.
